# The influence of paternal preconception health on infant birth weight: A scoping review

**DOI:** 10.1371/journal.pone.0344275

**Published:** 2026-03-13

**Authors:** Cindy-Lee Dennis, Karen McQueen, Justine Dol, Alistair Dennis-Grantham, Daisy R. Singla, Julia Carneiro Godoy de Sousa, Tina Montreuil, Catherine S. Birken, Shefaly Shorey

**Affiliations:** 1 Lawrence S. Bloomberg Faculty of Nursing, University of Toronto, Toronto, Canada; 2 Department of Psychiatry, Temerty Faculty of Medicine, University of Toronto, Toronto, Canada; 3 Lunenfeld-Tanenbaum Research Institute, Sinai Health, Toronto, Canada; 4 School of Nursing, Lakehead University, Thunder Bay, Ontario, Canada; 5 IWK Health, Halifax, Nova Scotia, Canada; 6 Campbell Family Mental Health Research Institute, Centre for Addiction and Mental Health, Toronto, Ontario, Canada; 7 Faculty of Nursing, Federal University of Goias, Goias, Brazil; 8 Department of Educational and Counselling Psychology, Faculty of Education, McGill University, Montreal, Quebec, Canada; 9 Research Institute of the McGill University Health Center, McGill University, Montreal, Quebec, Canada; 10 Department of Psychiatry, Faculty of Medicine and Health Sciences, McGill University, Montreal, Quebec, Canada; 11 Department of Pediatrics, Faculty of Medicine and Health Sciences, McGill University, Montreal, Quebec, Canada; 12 The Hospital for Sick Children, Toronto, Ontario, Canada; 13 Department of Paediatrics, Temerty Faculty of Medicine, University of Toronto, Toronto, Ontario, Canada; 14 Alice Lee Centre for Nursing Studies, Yong Loo Lin School of Medicine, National University of Singapore, Singapore; 15 Director Yeo Boon Kim Mind Centre, National University of Singapore, Singapore; 16 Editor-in-Chief, Midwifery (Elsevier), Exeter, United Kingdom; King Saud University Medical City, SAUDI ARABIA

## Abstract

Birth weight is an important determinant of infant growth and development associated with neonatal morbidity (e.g., respiratory distress, hypoglycemia) and mortality, as well as long-term health risks such as developmental delays and chronic conditions (e.g., asthma, type 2 diabetes) in later life. These adverse health outcomes are particularly concerning when infants are born small or large for their gestational age. A potential strategy to improve optimal birth weight is preconception care, with consistent evidence demonstrating a relationship between maternal preconception health and infant birth weight. However, little attention has been given to the influence of paternal preconception health on pregnancy outcomes. This scoping review aimed to capture the existing literature and highlight evidence gaps regarding associations between paternal preconception health and infant birth weight. We followed the Joanna Briggs Institute methodology and the Preferred Reporting Items for Systematic Reviews and Meta-Analysis extension for Scoping Reviews. The review considered studies that included men in the preconception period who identified as the parent of a child for whom infant birth weight outcomes were reported. Medline, PsycINFO, Embase, Scopus and CINAHL databases were searched to June 30, 2024. Two independent reviewers screened the titles/abstracts and full-text articles. Data extraction was performed independently by two researchers using a standardized form in Covidence. The data were synthesized narratively according to the paternal preconception health factors identified within the included studies (e.g., physical well-being, health behaviors, substance use, environmental exposures, mental health, and treatment effects). From 7,690 citations, 57 published studies were included in the review. Most studies were conducted in China (n = 18, 31.6%) or the United States (US) (n = 17, 29.8%) and used a cohort design (n = 54, 94.7%). Our review identified growing evidence that specific paternal preconception factors, such as physical well-being (e.g., BMI, physical health), certain medications (e.g., recreational drugs, sulfonylureas, diazepam), and environmental chemical exposure, may adversely influence infant birth weight. There are mixed findings related to other paternal health factors, including some health behaviors (e.g., nutrition, sleep, physical activity), substance use, and mental health. Consideration should be given to expanding preconception counseling and public health initiatives to include fathers, to improve paternal health and potentially reduce risks to offspring (e.g., birth weight). We also identified key areas where further research is required to advance knowledge in this field.

## Introduction

### Background

Birth weight is a key determinant of neonatal health and a strong predictor of both short- and long-term developmental outcomes such as growth stunting, lower IQ and mortality [1–3]. Concerns arise when infant birth weight falls on either end (low birth weight [LBW] or high birth weight [HBW]), as both are associated with short- and long-term risks [4]. Low birth weight is a global problem affecting more than 20 million infants annually. Although the highest rates of LBW are seen in low-and middle-income countries [5], it is also a substantial concern in high-income countries because it impacts child health outcomes [6]. Infants with LBW are 20 times more likely to die compared to normal birth-weight infants [3]. Additionally, infants with LBW face considerable morbidity, including neonatal complications and an increased risk of long-term cognitive, neurological, developmental, mental, and physical impairments [1,6,7].

High birth weight, known as macrosomia, is also a significant problem [8,9]. Excessive fetal growth can be associated with increased short- and long-term risks for the mother and the infant [2]. A systematic review of maternal and neonatal complications found that pregnancies with macrosomia had a higher risk of emergency caesarean section, postpartum hemorrhage, and obstetric injury [10]. For the neonate, there was an increased risk of shoulder dystocia, obstetric brachial plexus injury, and birth fractures. Additionally, HBW is associated with greater long-term child health risks, such as obesity and metabolic syndrome [2].

A wide range of maternal factors have been associated with LBW, including sociodemographic disadvantage, obstetric complications, chronic health conditions, lifestyle behaviors, and environmental factors [11–13]. As such, LBW is widely recognized as an important indicator of maternal health, nutrition, healthcare delivery, and poverty [1]. This is reflected in the varying prevalence rates of LBW among low-middle- and high-income countries. In contrast, HBW is predominately driven by metabolic and nutritional factors with maternal obesity, gestational diabetes mellitus, and excessive gestational weight gain consistently identified as the principal factors [4].

One potential strategy to promote optimal birth weight is preconception care [14,15]. Preconception care aims to improve an individual’s (male or female) health during their reproductive years by reducing risk factors, promoting healthy lifestyle behaviours, and increasing readiness for pregnancy [16]. Addressing risks before pregnancy is ideal because it allows time to change unhealthy behaviours, such as physical inactivity or smoking, and improve health before embryonic development [17]. Emerging research suggests that when preconception health interventions effectively increase health knowledge (e.g., nutrition, chronic disease management) and behavioural change (e.g., smoking, obesity), pregnancy and neonatal outcomes may improve [16–18].

While existing evidence demonstrates a robust relationship between maternal preconception health and infant birth weight [14,15], there has been a limited focus on how paternal preconception health influences outcomes. A recent review assessed modifiable paternal health variables and risk factors in the preconception period and found a trend that higher paternal BMI was associated with higher offspring birth weight [19]. The authors concluded that the positive associations between increasing paternal BMI and infant birth weight may be due to paternal sperm quality and potential changes in sperm epigenetic profiles, and that further research was warranted. A limitation of the review was that it focused on modifiable factors, and other factors, such as paternal health (e.g., comorbidities) and treatments (e.g., medications) were excluded. Paternal physical health, such as diabetes [20], metabolic syndrome [21], and some treatment medications [22] have been associated with sub-optimal birth weight outcomes. Similarly, paternal preconception exposure to some environmental or occupational toxins have been associated with lower birth weight [23,24]. However, several studies have found no association between these paternal variables and birth weight [25–27]. Emerging research suggests that paternal preconception health may impact fertility and the genetic contribution to offspring [28,29], thus a broader examination and synthesis of paternal factors is warranted. Due to the significant health risks associated with high and low birth weight [6,7,9], it is essential to examine paternal preconception health and infant birth weight to develop comprehensive preventative preconception care.

### Review objective

This review was conducted as part of HeLTI Canada (www.helticanada.com), an innovative preconception trial targeting child obesity [30]. The overarching scoping review was focused on the influence of paternal preconception health factors on perinatal and early childhood outcomes (see published protocol) [31]. Due to the large number of studies and varying outcomes in the full review, the specific objective of this review was to identify, consolidate, and synthesize the literature on paternal preconception health and infant birth weight.

## Materials and methods

### Design

This review followed the Preferred Reporting Items for Systematic Reviews and Meta-Analysis (PRISMA) extension for Scoping Reviews (PRISMA-ScR) [32] and the Joanna Briggs Institute (JBI) methodology for scoping reviews [33]. It also follows a published protocol [31]. Ethical approval was not applicable.

### Search strategy

A multi-step process was followed to identify published studies. First, the search keywords were refined and specified, and a preliminary search of the Web of Science was conducted. Second, assistance from an experienced health science librarian was sought to ensure the accuracy of the search keywords and the resulting search strategy (S1 Table). The final search strategy was adapted for each database, including MEDLINE All (Ovid), Embase (Elsevier), CINAHL Full Text (EBSCO), Scopus (Elsevier), and PsycINFO (EBSCO). We also conducted a manual search of the reference lists of included studies for additional articles, ensuring that no relevant articles were missed. The search was conducted on January 16, 2024 and updated on June 30, 2024.

### Inclusion and exclusion criteria

The review considered studies that included: [1] men who identified as the contributing reproductive partner for a child, [2] male preconception health exposure data, and [3] infant birth weight outcomes. Studies that solely reported on maternal exposures or did not separate paternal exposures were excluded. This review considered all English-language quantitative and qualitative studies (prospective and retrospective), including mixed methods. However, experimental studies evaluating intervention effectiveness were excluded because the review aimed to evaluate naturally occurring paternal preconception health characteristics rather than the effects of targeted interventions. Observational studies are therefore the most appropriate design for addressing our review question. Review articles, letters to the editor, editorials, commentaries, conference abstracts, dissertations, books, book chapters, and grey literature were also excluded to ensure that only peer-reviewed articles evaluating associations were included. Finally, only studies published from 2013 were included to synthesize the current evidence. The year 2013 was chosen because it reflected the past decade of research on the topic at the time the initial search was conducted.

#### Concept definition.

Birth weight is typically defined as the first weight of a newborn obtained after delivery. Within the included studies, birth weight was often categorized as: low birth weight (LBW) (less than 2,500 grams); normal birth weight (2,500–3,999 grams); or high birth weight (HBW) (4,000 grams or more). This classification reflects the newborn’s absolute birth weight, independent of gestational age. As such, some studies categorized birth weight by gestational age and SGA or LGA status.

### Study selection

All citations identified in the search were uploaded to Covidence [34], and duplicates were removed. Prior to the full review of title/abstracts, one reviewer piloted the inclusion/exclusion criteria to ensure that the list of reasons for exclusion was clearly outlined to minimize potential confusion and conflict among reviewers. After this pilot, two reviewers screened the titles and abstracts, then the full texts of potentially relevant studies, with disagreements resolved through discussion or by a third reviewer. Reasons for exclusion at the full-text stage are reported.

### Data extraction and synthesis

Data extracted from full-text articles included specific information, such as study design, sample size, participants, methods, paternal preconception exposures and measurements, and primary outcome results. Two reviewers extracted full data, which a third reviewer verified. Data were then organized and summarized by paternal preconception health exposures and infant birth weight. No quality appraisal was undertaken, consistent with the scoping review methodology [35].

## Results

### Search results

**Fig 1:**
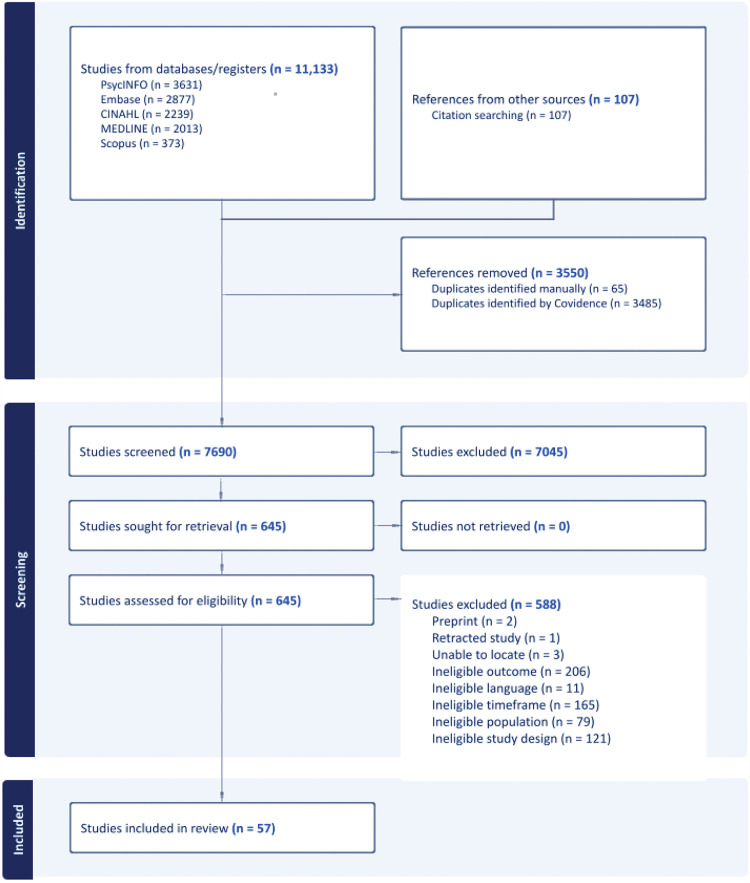
PRISMA flow diagram.

A total of 11,240 citations were retrieved from five databases and citation searching. After removing duplicates, the titles and abstracts of 7,690 citations were reviewed. Full-text screening was conducted for 645 studies, of which 57 were deemed eligible for inclusion. The search results and reasons for exclusion are reported in a PRISMA flow diagram (Fig 1). The most common reasons for exclusion were ineligible outcome (e.g., not infant birth weight), ineligible time frame (e.g., not pre-conception period), and ineligible study design (e.g., abstract, editorial).

### Study and population characteristics

All 57 included studies were published between 2013 and 2024, with 35 (61.4%) published in the last 5 years (Table 1). Most studies were conducted in China (n = 18, 31.6%) or the United States (US) (n = 17, 29.8%), with the remaining studies primarily from developed European countries (n = 16, 28.1%). Almost all were cohort studies (n = 54, 94.7%) that utilized diverse recruitment settings, including population-based samples identified through social media, large data sets, hospitals, and community clinics.

**Table 1:** Characteristics of included studies.

Author, Year (Country)	Study Design	Sample Size	Paternal Health Condition/Preconception Period	Control for Confounders (yes)	Birth Weight
**Physical Well-Being**					
**Body Mass Index (BMI)**					
Bibi et al., 2022 (Pakistan) [36]	Cross-sectional	750 fathers	Overweight > 24.5–30 kg/m2 during fertility treatment	None	The paternal overweight group was associated with a significant increase in infant birth weight.
Guo et al., 2022 (China) [37]	Retrospective cohort	4,719,813 fathers	Underweight < 18.5 kg/m2 Normal weight 18.5–25.0 kg/m2 Overweight 25.0–30.0 kg/m2 Obesity 30.0 kg/m2 or higher (every 3 months until 1 year after conception)	Paternal and maternal	Paternal BMI was associated with an increased risk of LGA. Paternal underweight is significantly predictive of a higher risk of SGA.
Kim et al., 2021 (US) [25]	Prospective cohort	1,889 fathers	Overweight 25–29.9 kg/m2 Obesity ≥30.0 kg/m2 (prior to IVF cycle)	Maternal only	Paternal BMI was not associated with birth weight.
Li et al., 2024 (China) [38]	Retrospective cohort	1,609 fathers	Overweight 25 kg/m2 ≤ BMI < 30 kg/m2 Obesity BMI ≥ 30 kg/m2 (SR)	Paternal and maternal	Paternal overweight was associated with an increased incidence of macrosomia. LGA was higher in the paternal overweight category.
Lin et al., 2022 (China) [39]	Prospective cohort	7,683 fathers	Overweight 24 ≤ BMI < 28 kg/m2 Obesity BMI ≥ 28 kg/m2 (SR)	None	Paternal obesity was associated with macrosomia and SGA. Paternal underweight BMI was also associated with SGA.
Lin et al., 2023 (China) [40]	Prospective cohort	29,518 fathers	Overweight 24 ≤ BMI < 28 kg/m2 Obesity BMI ≥ 28 kg/m2 (SR within 6 months of pregnancy)	Paternal and maternal	Paternal overweight and obesity groups were significantly associated with macrosomia.
Ma et al., 2020 (China) [41]	Multicenter prospective cohort	7,908 fathers	Overweight 23 to <27.5 kg/m2 Obesity ≥27.5 kg/m2 (SR during fertility treatment)	Paternal and maternal	Paternal overweight was associated with macrosomia. LGA was significantly higher in paternal overweight and obesity categories.
McPherson et al., 2021(Australia) [46]	Retrospective cohort	1,778 fathers	Underweight <18.5 kg/m2 Obesity class III > 40 kg/m2 (electronic scale prior to fertility treatment)	Paternal and maternal	Paternal preconception BMI did not predict birth weight.
Moss & Harris, 2015 (USA) [20]	Prospective Cohort	372 fathers	Overweight 25–29.9 kg/m2 Obesity ≥30 kg/m2 (interview measured height and weight)	Paternal and maternal	Paternal overweight and obesity categories were not significantly associated with birth weight.
Mutsaerts et al., 2014 (Netherlands) [47]	Population-based Prospective Cohort	2,264 fathers	Overweight 25.0–29.9 kg/m2 Obesity ≥30.0 kg/m2 (6 months prior to pregnancy	None	Paternal BMI was not significantly associated with SGA.
Noor et al., 2019 (US) [42]	Cohort	429 fathers	Overweight ≥ 25.0 kg/m2 (maternal report at study enrolment)	None	Paternal BMI was significantly associated with increased birth weight.
Retnakaran et al., 2021 (China) [48]	Prospective cohort	1,292 fathers	BMI (physical exam during pre-gravid recruitment time NR)	Paternal and maternal	Paternal preconception BMI was not independently associated with LGA or SGA.
Sun et al., 2022 (China) [49]	Multicenter prospective cohort	34,104 fathers	Overweight 23 to <27.5 kg/m2 Obesity ≥27.5 kg/m2 (physical exam and measurements; pre-pregnancy time NR)	Paternal and maternal	Paternal overweight or obesity was associated with increased incidence of LBW and very LBW.
Wei et al., 2022 (China) [50]	Prospective cohort	34,104 fathers	Overweight 24 to <27.9 kg/m2 Obesity ≥28 kg/m2 (SR pre- conception)	Paternal and maternal	Paternal overweight and obesity were associated with LBW and very LBW.
Xu et al., 2022 (China) [43]	Prospective cohort	1,810 fathers	Normal < 24.0 kg/m2 Overweight 24 kg/m2 < 28.0 kg/m2 Obese≥ 28.0 kg/m2 (SR pre-conception)	Paternal and maternal	Paternal preconception BMI was associated with increased offspring’s birth weight.
Yang et al., 2015 (China) [44]	Population-based case–control	6,341 fathers	Overweight 24 to <27.9 kg/m2 Obesity ≥28 kg/m2 (SR of pre-conception weight)	Paternal and maternal	Paternal overweight and obese BMI had an increased risk of macrosomia.
Zhang et al., 2023 (China) [45]	Cohort	2,075 fathers	Overweight 25–29.9 kg/m2 Obesity ≥30.0 kg/m2 (patients undergoing infertility treatment)	Paternal and maternal	Paternal BMI was associated with macrosomia, LGA, and “very” LGA.
**Physical Health**					
Fan et al., 2015 (China) [51]	Prospective cohort	1,017 fathers	Chronic disease (diagnosed by a physician pre-pregnancy)	Paternal and maternal	No association between paternal chronic disease and infant birth weight.
Kasman et al., 2020 (USA) [21]	Retrospective cohort	785,809	Metabolic syndrome (by medical records pre-conception)	None	Paternal comorbidity was associated with an increased odds of LBW infant.
Moss & Harris, 2015 (USA) [20]	Prospective cohort	372 fathers	Diabetes (SR pre-conception)	Paternal and maternal	Birth weight was significantly lower for infants born to fathers with diabetes.
Sun et al., 2021 (China) [52]	Population-based cohort	3,668,004 couples	Tuberculosis (SR pre-conception)	Paternal and maternal	There was no significant difference in infant birth weight for fathers with and without tuberculosis.
**Health Behaviours**					
**Nutrition**					
Martín-Calvo et al., 2019(USA) [53]	Cohort	108 fathers	Total folate intake (Dietary Folate Equivalents) (S-R)	Paternal and maternal	Paternal preconception folate intake was not associated with birth weight or SGA.
Moss & Harris, 2015 (USA) [20]	Cohort	372 fathers	Frequency of fast-food consumption (# days/week) (SR)	Paternal and maternal	There was no association between the frequency of fast-food consumption and birth weight.
**Sleep Quality**					
Du et al., 2022 (China) [54]	Retrospective cohort	282 fathers	Sleep quality (Pittsburgh Sleep Quality Index when receiving fertility treatment)	Paternal and maternal	There was no significant difference in birth weight and sleep groups (poor or good).
**Physical Activity**					
Moss & Harris, 2015 (USA) [20]	Cohort	372 fathers	Physical activity (8-item SR questionnaire)	Paternal and maternal	There was no association between the frequency of physical activity bouts and birth weight.
Mutsaerts et al., 2014 (Netherlands) [47]	Population-based Prospective Cohort	2,264 fathers	Physical activity (SR)	Paternal and maternal	There was no association between the father’s level of physical activity and birth weight.
**Substance Use**					
**Recreational Drugs**					
Lin et al., 2021 (Taiwan) [55]	Population-based cohort	18,678 fathers	At least one Drug Criminal Record of a schedule I or II drug within 2 years of pregnancy	Paternal and maternal	Paternal preconception use of illegal drugs was significantly associated with LBW.
Moss & Harris, 2015 (USA) [20]	Prospective cohort	372 fathers	Use of marijuana or other drugs (SR in the last year)	Paternal and maternal	Paternal marijuana or other drug use was not associated with offspring birth weight.
**Smoking**					
Fan et al., 2015 (China) [51]	Prospective cohort	1,017 fathers	Smoking (SR yes/no)	Paternal and maternal	Paternal smoking was not associated with birth weight.
Horwitz et al., 2022 (China) [56]	Prospective cohort	1,174 fathers	SR smoking cigarettes (no smoking, 1–0/day, > 10/day)	Paternal and maternal	Paternal smoking was not associated with birth weight.
Ko et al., 2014 Taiwan [57]	Prospective longitudinal cohort	NR	SR smoking (before, during, or 4 months after pregnancy [no smoking, 1–0/day, > 10/day])	Paternal and maternal	Paternal smoking was not associated with LBW or SGA.
Moss & Harris, 2015 (USA) [20]	Prospective cohort	372 fathers	SR smoked at least one cigarette per day over the last 30 days	Paternal and maternal	Paternal smoking was not associated with birth weight.
Mutsaerts et al., 2014 Netherlands [47]	Population-based prospective cohort	2,264 fathers	Daily smoking (SR # of cigarettes/day)	None	Paternal smoking was not associated with SGA.
**Alcohol**					
Fan et al., 2015 (China) [51]	Cohort	829 children with parent data	Drinking (SR yes/no)	Paternal and maternal	Paternal alcohol consumption was not associated with birth weight.
Moss & Harris, 2015 (USA) [20]	Prospective cohort	371 fathers	SR frequency of alcohol use (once per month or less/more)	Paternal and maternal	No significant difference was found regarding infant birth weight with paternal alcohol use.
Mutsaerts et al., 2014 (Netherlands) [47]	Cohort	2,264 couples	SR paternal alcohol consumption (units/week)	Paternal and maternal	Paternal alcohol consumption was not associated with infants being SGA.
Xia et al., 2018 (China) [58]	Cohort	980 infants/ parent data	SR paternal alcohol 1/week at 3 months before conception	Paternal and maternal	Paternal alcohol use was not associated with LBW.
**Environmental Exposures**					
**Persistent Organic Pollutants**					
Bloom et al., 2015 (USA) [23]	Cohort	501 fathers	Presumed exposure to persistent organic pollutants (urine and blood up to 12 months prior to conception)	Paternal and maternal	Lower birth weight was associated with greater paternal exposure. Paternal urine Arsenics were linked to higher birth weight.
Robledo et al., 2015 (USA) [59]	Cohort	234 fathers	Persistent organic pollutants (blood)	Paternal and maternal	Several persistent organic pollutants were associated with lower birth weights
**Phthalates**					
Messerlian et al., 2017 (USA) [24]	Cohort	195 fathers	Phthalate exposure (urine)	Paternal and maternal	Paternal preconception urinary concentration of the sum of DEHP metabolites was associated with a decrease in birth weight.
Mustieles et al., 2019 (USA) [60]	Cohort	68 fathers	Phthalate exposure in urine obtained during fertility treatment	Paternal and maternal	Paternal DEHP metabolite concentrations were not associated with birth weight: placenta weight
Smarr et al., 2015 (USA) [61]	Cohort	233 fathers	BPA, phthalates, and creatinine (blood and urine)	Paternal and maternal	Paternal urinary concentration of BPA and specific phthalate metabolites may be associated with a reduction in birth weight.
Zang et al., 2023 (China) [62]	Cohort	1,484 fathers	17 phthalate metabolites measured (urine during fertility treatment)	Paternal and maternal	Various phthalate metabolites were associated with an increased risk of an LBW infant
Zhang et al., 2022 (USA) [63]	Cohort	211 fathers	Urinary concentrations of 4 phenols and 11 phthalates (blood and urine during fertility treatment)	Paternal and maternal	Low-molecular-weight phthalates and DEHP-BPA factors were associated with reduced birth weight.
**Phenols**					
Messerlian et al., 2018 (USA) [64]	Cohort	184 fathers	Phenol exposure (at least one urine sample pre-conception)	Paternal and maternal	There was a positive association between paternal preconception benzophenone-3 and birth weight.
Mustieles et al., 2018 (USA) [26]	Cohort	190 fathers	Bisphenol exposure BPA and BPS concentrations (urine)	Paternal and maternal	No associations between paternal preconception urinary BPA concentrations and birth weight.
**Other Chemicals**					
Redmond et al., 2022 (USA) [65]	Cohort	155 fathers	Participants with elevated background levels of PCB (blood)	Paternal and maternal	The findings suggest that increased paternal PBB and PCB levels negatively impact birth weight.
Zhang et al., 2024 (USA) [66]	Cohort	145 fathers	Nine PFAS (serum during fertility treatment)	Paternal and maternal	Paternal preconception concentrations of PFAS were associated with higher birth weight.
**Radiation**					
Chen et al., 2018 (China) [67]	Cohort	192,492 fathers	Radiation (SR x-ray, CT scan, etc. yes/no 6 months prior to conception)	Paternal and maternal	Paternal history of radiation exposure was related to LBW.
**Mental Health**					
**Depression**					
Kasman et al., 2020 (USA) [21]	Retrospective cohort	785,809 live births	Depression (diagnosis by diagnostic codes)	None	Increased odds of LBW if the father had depression.
Moss & Harris, 2015 (USA) [20]	Prospective cohort	372 fathers	Depression (SR scale)	Paternal and maternal	No association between paternal depression and infant birth weight.
Spry et al., 2020 (Australia) [68]	Cohort	267 fathers	Common mental disorder symptoms (SR history of depression, anxiety, psychological distress)	Paternal and maternal	Little evidence of an association between paternal preconception mental disorder symptoms and SGA.
**Treatment Effects**					
**Medication Use**					
Boyer et al., 2020 (France) [69]	Cross-sectional	1,332 fathers	Immunosuppressant (SR at the time of conception)	Paternal only	6% had low birth weight (< 2.5 Kg).
Engeland et al., 2013 (Norway) [70]	Cohort	349,020 fathers	Special attention drug (prescription 3 months before pregnancy)	Paternal and maternal	Increased odds of being SGA when the father was dispensed diazepam prior to conception.
Friedman et al., 2022 (Denmark) [71]	Cohort	6,976 fathers	Anti-inflammatory/ Immunosuppressive (3 months before conception)	Paternal and maternal	No increased risk was identified.
Jones et al., 2013 (USA) [72]	Cohort	152 fathers	Mycophenolic acid products (SR prior to conception)	None	4.1% were LBW (<2500 grams), lower than the general population rate.
Larsen et al., 2016 (Dennark) [73]	Cohort	399,870 fathers	Anti-TNF-α agents (within 3 months before conception)	Paternal and maternal	Risk of SGA was not statistically significant.
Larsen et al., 2018 (Denmark) [74]	Cohort	101,3994 fathers	Systemic corticosteroids (within 3 months before conception)	Paternal and maternal	Increased risk not statistically significant.
Lund et al., 2023 (Dennark) [75]	Cohort	126,0934 fathers	Non-steroid anti-inflammatory, opioids within 3 months of conception	Paternal and maternal	No clinically important associations.
Meserve et al., 2021(USA) [27]	Cohort	7,453 fathers	Immunosuppressive or biologic (database)	Paternal and maternal	There was no association between paternal exposure to any immunosuppressive or biologic agent and LBW.
Morken et al., 2015 (Norway) [76]	Cohort	2,463 fathers	Immunosuppressive (group before and after solid organ transplantation)	Paternal and maternal	No significant difference in SGA.
Nørgård et al., 2017 (Denmark) [77]	Cohort	101,3323 fathers	Azathioprine/6-mercaptopurine (within 3 months before conception)	Paternal and maternal	No statistically significant increased risk of SGA.
Nørgård et al., 2022 (Denmark) [78]	Cohort	9,900 fathers	5-aminosalicylic acid (3 months before conception)	Paternal and maternal	Children did not have increased SGA.
Nørgård et al., 2022 (Denmark) [22]	Cohort	7,648 fathers	Antidiabetic drugs (prescription for drug 3 months before conception)	Paternal and maternal	Increased risk of SGA for sulfonylureas. Not significant for insulin or metformin and SGA.
Uyaroglu, 2020 (Turkey) [79]	Cohort	45 fathers	Anti-TNF alpha agents (SR use before pregnancy)	None	Of 39 live births, 6 (15.4%) had LBW.
VanderMijle et al, 2023(Netherlands) [80]	Cohort	15,959 fathers	Immunosuppressants (3 months before conception)	Paternal and maternal	Six children (11.1%) were SGA.
Wallenius et al., 2015 (Norway) [81]	Cohort	1,796 fathers	Disease-Modifying Antirheumatic Drug (DMARD) (database information of medication 12 weeks before conception)	Paternal and maternal	Mean differences in birth weights were not different in the DMARD-exposed group and the never DMARD-exposed group.
Weber-Schoendorfer et al., 2014 (Germany) [82]	Cohort	525 fathers	Low-dose methotrexate (SR within 3 months prior to conception)	None	There was no significant difference in birth weight between groups.
Winter et al., 2017 (Denmark) [83]	Cohort	101,3994 fathers	Methotrexate (within 3 months before conception)	Paternal and maternal	No increased odds of SGA.
Zarén et al., 2023 (Sweden) [84]	Cohort	809,929 fathers	Methotrexate (prescription codes)	Paternal and maternal	No difference between groups for medication exposure and SGA.

BMI: body mass index; BPA: Bisphenol A; PBB: polybrominated biphenyl; BPS: Bisphenol S; DEHP: di(2-ethylhexyl) phthalates; LBW: low birth weight; LGA: large for gestational age; PCB: polychlorinated biphenyl; PFAS: polyfluoroalkyl substances; NR: not reported; SGA: small for gestational age; SR: self-report

### Data synthesis

The paternal preconception health categories identified in this review included physical well-being, health behaviours, substance use, environmental exposures, mental health, and treatment effects. The reporting of infant birth weight was highly variable. Many studies operationalized birth weight by gestational age (LGA or SGA); others reported infant weight as LBW, macrosomia (>4000 grams; yes/no) or in specific grams. For our review, we utilized the definitions provided in the studies.

### Physical well-being

#### Body mass index (BMI).

Seventeen studies reported on the influence of paternal BMI on infant birth weight. While the findings were mixed, most (n = 12 of 17, 70.6%) identified a significant association (Table 2).

**Table 2:** Synthesis of significant associations.

Preconception Health Factor	Number of Studies Included	Association (yes) Number of studies	Notes
**Physical Well-Being**			
BMI	17	12	Both high and low BMI were associated with suboptimal birth weight (low or high).
Physical Health	4	2	Comorbidities (metabolic syndrome and diabetes) were associated with lower birth weight.
**Health Behaviors**			
Nutrition	2	0	NS
Sleep quality	1	0	Equivocal
Physical activity	2	0	NS
**Substance Use**			
Recreational drugs	2	1	Equivocal (except for illegal drugs and LBW)
Smoking	5	0	NS
Alcohol	4	0	NS
**Environmental Exposures**			Diverse environmental exposures were associated with lower birth weights.
Organic pollutants	2	2	
Phthalates	5	4	
Phenols	2	1	
Other chemicals	2	2	
Radiation	1	1	
**Mental Health**			
Depression	3	1	NS in most studies
**Treatment Effects**			NS (except for sulfonylureas and diazepam associated with SGA)
Medication Use	18	2	

*NS: not significant.*

#### Paternal preconception BMI and HBW.

In 10 studies, researchers reported paternal BMI was associated with increased infant weight [36–45]. Two large cohort studies conducted in China found that paternal preconception overweight and obesity were significantly associated with macrosomia (p = 0.038 and *p* < 0.001, respectively) [40], with infants of obese fathers 1.7 times more likely to be macrosomic at birth (95%CI: 1.168, 2.444) [39]. Similar results were found in a case-control study of 6,341 Chinese fathers, where infant macrosomia was higher among fathers who were overweight (aOR=1.33; 95%CI: 1.11,1.59) and obese (aOR=1.99; 95%CI: 1.49, 2.65) [44]. In a retrospective cohort study of 4.7 million Chinese couples, overweight (OR=1.08; 95%CI: 1.06, 1.09) and obese (OR=1.19; 95%CI: 1.17, 1.20) fathers had a significantly increased odds of LGA infants, while underweight fathers had a significantly higher odds (OR=1.17; 95%CI: 1.15, 1.19) of SGA infants compared with fathers with normal BMI [37], after adjusting for potential covariates. Three additional Chinese cohort studies reported multiple HBW outcomes. One large study (n = 2,075 fathers) found paternal overweight status to be associated with LGA (aOR=1.70; 95%CI: 1.05, 2.74) and paternal obesity to significantly increase the likelihood for very LGA (aOR=2.58; 95%CI: 1.03, 6.45) and macrosomia (aOR=3.28; 95%CI: 1.33, 8.09) [45]. Two additional studies with 7,908 [41] and 1,609 [38] fathers confirm paternal preconception overweight and obesity significantly increase the odds for LGA and macrosomia compared to normal BMI controls after adjusting for confounders.

Three studies found a positive relationship between paternal BMI and increased birth weight [36,42,43]. In a cross-sectional study of 750 Pakistani fathers [36], those who were overweight had infants with significantly higher birth weights (2952.14 ± 53.64g) when compared with those in the non-overweight group (2577.24 ± 30.94g). In a longitudinal US cohort study with 429 fathers, paternal BMI (≥25.0 kg/m2) was significantly associated with increased birth weight compared to those with a BMI < 25 (mean [SD] z score, 0.38 [0.91] vs 0.11 [0.96]; p = .004) [42]. Lastly, in a cohort of 1,810 Chinese fathers, each standard deviation increment of paternal BMI was associated with an additional increase in birth weight (aOR=29.6; 95%CI: 5.7, 53.5) that was sex-specific to male infants only [43].

### Paternal preconception BMI and low infant birth weight

Three studies in China reported an association between increased paternal preconception BMI and low infant birth weight [39,49,50]. A prospective cohort study that included 7,683 fathers found that paternal obesity was significantly associated with SGA infants (SGA; OR=2.866; 95%CI: 2.091, 3.930), while paternal low BMI (underweight) was also associated with SGA in infants (OR=8.506; 95%CI: 5.784, 12.509) [39]. Paternal pre-pregnancy BMI (n = 34,104) was associated with LBW among overweight (aOR=1.50; 95%CI: 1.36, 1.64) and obese (aOR=1.36; 95%CI: 1.19,1.54) fathers [49]. Very LBW was also associated with overweight (aOR=1.31; 95%CI: 1.10, 1.56) and obese (aOR=1.31; 95%CI: 1.04, 1.65) fathers. Similarly, paternal BMI (n = 34,104 fathers) was identified as a risk factor for LBW (OR=1.346; 95%CI: 1.117, 1.622) among fathers who were overweight [50].

### Paternal preconception BMI and no association with infant birth weight

Five studies reported no associations between paternal preconception BMI and birth weight [20,25,46–48]. In the first US study that included 1,889 fathers, there was no statistically significant difference in infant birth weight among paternal BMI groups in the overweight, obesity, and normal categories [25]. In the other US study (n = 372 fathers), paternal overweight (OR=35.6; 95%CI: −140.0, −211.3) and obesity (OR=76.8; 95%CI: −74.6, −228) were not significantly associated with infant birth weight [20]. Data from 2,264 in a prospective population-based birth cohort found paternal BMI was also not associated with SGA (OR=0.96; 95%CI: 0.91, −1.01) [47]. Similar results were found in a sample of 1,292 fathers from China [48] and 1,778 fathers from Australia [46].

### Physical health

Four studies examined the relationship between paternal physical health and birth weight [20,21,51,52]. Two studies conducted in the US noted an association between paternal physical health and infant birth weight. In the first large cohort study (n = 785,809 live births), paternal comorbidity with all or most components of metabolic syndrome was associated with 23% higher odds of an infant with LBW (95%CI: 1.01, 1.51) [21]. In the study by Moss and Harris [20], researchers found that birth weight was lower among infants born to fathers with diabetes compared to those born to fathers without the condition (difference = −783.9; 95%CI: 1014.2, 553.6, p < 0.01). Two studies in China found no association between infant birth weight and fathers with chronic disease [51] andsupp tuberculosis [52].

### Health behaviours

#### Nutrition.

Two studies investigated the impact of paternal preconception nutritional status on birth weight [20,53]. A cohort study in the US found no significant relationship between paternal folate intake and birth weight or risk for SGA after multivariable adjustments [53]. Likewise, Moss and Harris [20] evaluated the frequency of fathers’ fast-food consumption and found no association between this dietary pattern and birth weight.

#### Sleep quality.

In a retrospective cohort study, researchers evaluated the association between paternal sleep quality and birth outcomes among 282 couples undergoing ART in China [54]. While there was no statistically significant difference in birth weight between the poor and good sleep groups, multivariate analysis revealed that the global sleep quality score negatively influenced birth weight (β=−63.81; 95%CI: 119.91, 8.52, p = 0.047).

#### Physical activity.

Two studies examined the influence of paternal physical activity on birth weight [20,47]. In a US cohort study, Moss and Harris [20] investigated the frequency of activity bouts in the past week and found no significant association with birth weight. Similarly, in the population-based cohort study, Mutsaerts et al. [47] assessed physical activity levels, defining positive activity as engaging in moderate-intensity exercise for at least 30 minutes at least once a week. Their results showed no independent association between paternal physical activity levels and birth weight.

### Substance use

#### Recreational drugs.

One population-based retrospective cohort study (n = 18,678) linked to the national database in Taiwan demonstrated that paternal preconception use of illegal drugs within one year (−115.05g, 95%CI: −223.89, −6.21) or two years (−128.33g, 95%CI: −254.05, −2.6) was significantly associated with LBW compared to unexposed infants [55]. In a smaller US prospective cohort study, paternal use of marijuana or other drugs within the last year was not associated with birth weight [20].

#### Smoking.

Five studies examined paternal preconception smoking and birth weight outcomes, and none demonstrated a direct or independent effect on birth weight outcomes [20,47,51,56,57]. A large longitudinal cohort study in Taiwan investigated paternal smoking during different stages of pregnancy with birth weight. They found that paternal preconception smoking was not associated with LBW or SGA [57]. Likewise, Mutsaerts [47] found no association between paternal daily smoking and SGA. The three remaining studies evaluated paternal smoking over the last 30 days [20], smoking dichotomized as yes/no [51] and smoking 1–10 cigarettes/day vs > 10/day [56] and found no association with birth weight.

#### Alcohol.

Four cohort studies analyzed paternal preconception alcohol use and birth weight [20,47,51,58]. These studies found that paternal alcohol consumption was not associated with infant birth weight. In one US study [20], alcohol use was dichotomized to drinking once per month or less compared to more frequently. No significant difference was found regarding birth weight in the bivariate analysis with paternal alcohol use. In another study [47], data on paternal alcohol consumption (units per week) were collected with univariable and multivariable logistic regression analyses suggesting paternal alcohol use did not independently influence SGA. Likewise, two cohort studies conducted in China with 829 infants [51] and 980 infants [58] examined paternal alcohol use (yes/no) and LBW at birth and found no differences between groups.

### Environmental exposures

#### Persistent organic pollutants.

Two cohort studies in the US evaluated paternal preconception exposure to various persistent organic pollutants and their impact on birth weight [23,59]. In the first study, higher paternal urinary levels of cesium (β = −237.85, 95%CI −463.04, −12.66), uranium (β = −187.34, 95%CI: −366.34, −8.35), zinc (β = −209.08, 95%CI: −417.40, −0.77) and chromium (2nd tertile: β = −140.52, 95%CI: −317.40, 36.35, 3rd tertile: β = −144.33, 95%CI: −343.28, 54.62) were associated with lower birth weight [23]. Conversely, a higher paternal urinary arsenic level was associated with higher birth weight (β = 194.71, 95%CI: 17.13, 372.30). In the other study, researchers evaluated paternal preconception exposure to 63 persistent organic pollutants among 234 infants [59]. Serum concentrations of polychlorinated diphenyl ether-183 and polychlorinated biphenyls-167 were associated with lower birth weight among 116 girls [(β = –92.13 g; 95%CI: –173.44, –10.82) and (β = –97.49 95%CI: –187.45, –7.54), respectively]. Among boys (n = 113), serum concentrations of several persistent organic pollutants were also associated with lower birth weight (range, 98–170 g).

#### Phthalates.

Five cohort studies evaluated paternal preconception exposure to phthalates, with four studies finding an association between paternal phthalate exposure and birth weight [24,60–63]. In a US cohort of 195 fathers, researchers found a significant negative association between paternal urinary concentration of one metabolite (sum of di[2-ethylhexyl]) phthalate (ΣDEHP) and birth weight [24]. Another US study evaluated paternal preconception urinary concentrations of 14 phthalate metabolites and infant birth weight [61]; only paternal monoethylhexyl phthalate (mEHP) was associated with a 191.93 g decrease in birth weight (95%CI: −381.61, −2.25). A third US study examined phenol and phthalate metabolite exposure to birth weight among offspring born to sub-fertile couples [63]. Multivariable linear models revealed a 63g (95%CI:–134, 7) and 73g (95%CI: –141, –5) decrease in birth weight was associated with each unit increase in paternal scores of the DEHP metabolites-BPA factor and low molecular weight phthalate factor, respectively. In a study in China [62], higher paternal prenatal concentrations of mono-benzyl phthalate (aRR = 1.40; 95%CI:1.04, 1.87) and mono-carbocisononyl phthalate (aRR = 1.53; 95%CI: 1.12, 2.09) were associated with an increase in LBW risk among spontaneously conceived offspring. Among ART-conceived offspring, the adjusted relative risk was lower for LBW with exposure to MnBP, mono-3-carboxypropyl (MCPP), mono-2-ethylhexyl (MEHP), and mono-2-ethyl-5-hydroxyhexyl (MEHHP). Finally, the fifth US study did not find a significant association between paternal DEHP concentrations and the birth weight: placental weight ratio [60].

#### Phenols.

Two US cohort studies evaluated paternal urinary phenol concentrations [26,64]. In the first study, researchers evaluated paternal preconception urinary phenol levels and birth weight among 346 single offspring [64]. For each log-unit increase in paternal preconception benzophenone-3 concentration in adjusted models, birth weight increased significantly by 137g (95%CI: 60, 214). In the second study, researchers evaluated paternal preconception urinary bisphenol concentration and birth weight (n = 346 infants) and found no association [26].

#### Other chemicals.

One US cohort study examined the relationship between birth weight and paternal preconception serum concentrations of polychlorinated biphenyl (PCB) and polybrominated biphenyl (PBB). In the US cohort study (n = 336 infants), the adjusted models revealed that an increase in paternal preconception serum concentrations of PCB and PBB were associated with increased risks of lower birth weight [65]. Another US cohort study evaluated paternal preconception serum concentrations of nine perfluoroalkyl and polyfluoroalkyl substances (PFAS) and birth weight among 312 infants [66]. Paternal preconception serum concentrations of perfluorooctanesulfonate (β:147.81g, 95%CI: − 7.90, 303.52) and perfluorohexanesulfonate (β:127.13g, 95%CI: −2.75, 257.00) were associated with higher birth weight; however, the relationships may have been imprecise due to the small sample size (n = 145) [66].

#### Radiation.

One cohort study in China evaluated paternal exposure to radiation (e.g., x-ray, CT, PET-CT) (n = 192,492) and birth weight [67]. Researchers found that paternal preconception exposure to radiation was associated with LBW (OR=1.54; 95%CI:1.08, 2.18). However, the average birth weight of infants in both the exposed and unexposed groups was within normal limits.

### Mental health

#### Depression.

Three studies examined the association between paternal mental health and birth weight with mixed findings [20,21,68]. A large US cohort study using diagnostic codes from inpatient and outpatient records found that fathers with a history of depression the year before pregnancy had an increased odds of having a LBW infant (OR=1.18; 95%CI:1.12, 1.24) [21]. Conversely, no associations were observed among 421 men with persistent preconception common mental disorder symptoms and SGA [68]. Likewise, Moss and colleagues [20] found no association between paternal depression and birth weight. The researchers also noted a low degree of participants with depression within their study.

### Treatment effects

#### Medication use.

Eighteen studies evaluated the birth weight of infants fathered by men with various health conditions receiving treatments in the preconception period [22,27,69–84]. Most studies consistently identified that paternal preconception medications were not associated with an increase in SGA or LBW infants. The studies included fathers with various health conditions such as transplants, chronic inflammatory conditions and diabetes. The fathers received a variety of treatments during the preconception period, including immunosuppressive agents [69,72,76,80], biological agents (e.g., tumour necrosis factor (TNF)-alpha inhibitors) [27,73,79], anti-inflammatory agents [74,78], antidiabetic drugs [22], thiopurines [77], disease-modifying anti-rheumatic drugs (DMARDs) [81], methotrexate [82–84], and other medications [70,71,75].

Two large cohort studies found an association between paternal medication use and increased SGA [22,70]. A Danish study evaluating antidiabetic agents and birth weight found an increased risk of SGA infants among fathers who were receiving sulfonylureas 3 months before conception (aOR=1.90; 95%CI:1.11, 2.93) compared with controls [22]. However, other antidiabetic agents such as insulin and metformin were not associated with SGA. Similarly, a Norwegian study found an increased risk of SGA among infants whose fathers were prescribed diazepam [70]. In this large cohort study (n = 349,020 fathers), researchers assessed the relationship between specific drugs dispensed to fathers within 3 months before conception and SGA. They observed no increased risk for SGA infants when fathers were prescribed any drug, drugs requiring special attention, or prednisolone. Nevertheless, among the 178 exposed infants to paternal diazepam, there was an increased risk of SGA (OR=1.4; 95%CI:1.2, 1.6).

## Discussion

This comprehensive review offers growing evidence on the link between paternal preconception health and infant birth weight, a significant public health concern because of its strong connections to neonatal morbidity, childhood development, and adult chronic disease risk [5]. Although maternal health has historically been the focus of preconception research and clinical guidelines, this evidence emphasizes that paternal factors—such as body composition, chronic conditions, some medications, and environmental exposures—also significantly influence early developmental processes in offspring.

The most frequently studied exposure was paternal preconception BMI, with most studies finding significant links between higher BMI and increased infant birth weight, including LGA and macrosomia. These links were especially strong in large Chinese cohort studies [40,41], with some showing dose-response relationships where increasing BMI was associated with higher offspring birth weight. However, some studies also reported increased risks of SGA and LBW with higher paternal BMI, suggesting nonlinear associations. These findings are not contradictory; rather, they highlight the complexity of the relationship and the likely presence of multiple underlying mechanisms that contribute to infant birth weight. Emerging evidence from human and animal studies on paternal obesity and epigenetic programming indicates that excess fat may affect offspring birth outcomes by altering sperm DNA methylation, histone modifications, or small non-coding RNAs [85,86]. Additionally, there is some evidence that paternal obesity may be related to impaired placental growth and suboptimal placental functioning, leading to growth restriction [39]. Lastly, the relationship between paternal BMI and infant birth weight should be considered alongside maternal risk factors and infant birth weight (e.g., nutrition, pre-existing medical conditions, and BMI) [4,11,12].

Five studies found no significant association between paternal BMI and infant birth weight, which may be due to heterogeneity across the included studies. There may be geographic and ethnic differences among participants in the studies from North America, Asia and Europe. For example, some Chinese studies used lower BMI cutoffs for overweight (BMI 24–28) and obesity (> 28) than the US studies (overweight: 25–29.9; obesity: > 30). Likewise, in most studies, the analyses controlled for potential confounding maternal and paternal variables; however, the adjustments were inconsistent across studies. Many studies fully adjusted for maternal and paternal factors, such as age, height, BMI, ethnicity, education, pregnancy complications, smoking, and alcohol [37], while others were limited by the available data and adjusted for only maternal [25] or no potentially confounding factors [36,39,42,47]. Lastly, variation exists with the outcome of birth weight, measured as a continuous variable versus dichotomous LGA (yes/no) or SGA (yes/no). These findings highlight the need for rigorous research in this area with attention to the influence of population-specific factors, the importance of adjusting for maternal variables, and differences in BMI and outcome measurements.

Our review also found increasing evidence that suggests that paternal physical health issues, especially metabolic syndrome and diabetes, are linked to a higher risk of LBW in offspring. This aligns with research indicating that paternal conditions may contribute to low birth weight by influencing fetal development and placental health [21]. This may also partially explain the relationship between high BMI and LBW if fathers with high BMI also have co-morbid conditions. However, not all chronic diseases showed associations, as no link was found with tuberculosis or other chronic conditions in two Chinese cohorts [51,52]. These null results may reflect differences in disease severity, management, or limitations in sample size.

Evidence regarding the role of paternal nutrition, physical activity, and sleep quality remains limited and largely inconclusive. While one study found a significant inverse relationship between sleep quality and birth weight [54], most other studies did not identify statistically significant links. With growing recognition of the biological significance of paternal lifestyle factors on sperm epigenetics [87], future research using more precise exposure assessments and longitudinal data is needed. Paternal preconception lifestyle factors, including substance use—particularly tobacco and alcohol—were not consistently associated with infant birth weight. This finding is somewhat unexpected given the known adverse effects of these substances on sperm quality and DNA integrity [88]. However, one large Taiwanese study linked paternal illegal drug use within one to two years prior to conception to lower birth weights, underscoring the need for more detailed exposure data and differentiation between substance types, usage frequency, and timing relative to conception.

Research on environmental exposures and their impact on birth outcomes suggests that paternal urinary or serum phthalate levels, as well as persistent exposure to organic pollutants (POPs) and heavy metals, may be associated with lower birth weight. Research has identified that diverse paternal environmental exposures can interfere with sperm DNA damage, leading to epigenetic abnormalities [89,90]; however, studies specific to the outcome of birth weight are lacking and warrant further mechanistic investigation. Additionally, our review found chemicals such as arsenic and PFAS have been linked to higher birth weights, highlighting the complex nature of dose-response relationships and the potential for non-monotonic effects. Only one investigation examined paternal ionizing radiation exposure and found an association with low birth weight [67]. Although the birth weights remained within normal ranges, this observation warrants further research, particularly given the increasing use of medical imaging and occupational radiation among men of reproductive age.

The link between paternal depression and birth weight produced mixed results. A large US cohort found increased odds of LBW in fathers with recent depression diagnoses, but smaller studies did not verify this association [21]. This discrepancy could be due to variations in mental health assessment methods (diagnostic codes versus self-report), symptom severity, or unmeasured factors like maternal mental health and family stress. Likewise, most research on paternal medication use for chronic conditions (like DMARDs, immunosuppressants, or steroid medications) found no association with LBW or SGA. Nevertheless, two large cohort studies noted increased SGA risks linked to paternal use of sulfonylureas and diazepam, hinting at drug-specific effects that need further investigation. These insights are clinically important because paternal medication use is often overlooked in reproductive counseling and risk assessment evaluations. However, further evaluation is still required to confirm the safety or risks of paternal preconception medications. Paternal medication exposures were often assessed through self-reports at a single time point, which may miss changing or cumulative impacts. Several studies evaluated pharmacy databases and prescriptions filled (yes/no), which do not consider dosage or length of administration.

### Implications for clinical practice and future research

The findings of the present review have important implications for preconception care and future research. First, given the influence of various paternal health factors on infant birth weight, greater male involvement in preconception care is warranted. Healthcare professionals and public health officials should consider factors such as paternal BMI, chronic medical conditions, medication use and environmental exposures rather than focusing mainly on maternal factors. Proper management could improve long-term health outcomes not only for the infant but also for potential parents. Where possible, healthcare providers should also inform individuals about risks and offer alternatives to minimize exposure to harmful chemicals. Identifying the specific components of paternal health that may impact infant birth weight also provides insight into developing interventions to mitigate risk and reduce adverse effects on fetal development. Lastly, this scoping review highlighted specific research gaps and limitations that warrant further investigation.

### Strengths and limitations

One of the strengths of this scoping review is the application of the JBI scoping review framework in the methodology and the published protocol to guide the review. Furthermore, a team-based approach combining expertise from clinical and research backgrounds was used, strengthening the planning and execution of this scoping review. Another key strength lies in the inclusion of diverse study populations and methodologies, thereby increasing the relevance of the results. However, this review also notes several limitations. The studies in this review were primarily conducted in the USA and China, which may not be generalizable to populations in other regions. The samples were also diverse, with some being population-based, others recruited from clinics (fertility and non-fertility), and others from insurance or pharmacy databases, which could lead to selection bias. Another limitation is that certain paternal health conditions, medications, or behaviours were uncommon and accounted for only a small portion of the review’s sample. Therefore, despite having a larger sample size, the power of the findings remains low because few individuals report having these conditions or engaging in certain behaviours. Some studies using large datasets recorded only live births. Thus, infants who may have been compromised in utero and miscarried or were stillborn would not be captured. As our review was specific to paternal preconception health and birth weight, the influence of combined paternal and maternal factors was not considered and remains an area for further research, as does the influence of paternal preconception health factors on other pregnancy and neonatal outcomes. Finally, the studies included showed heterogeneity, which limits comparability between them. As is customary with scoping reviews, we did not conduct quality appraisals of the included studies. However, we have highlighted the limitations of the existing evidence and potential sources of bias (e.g., sample size and selection, various exposure measurement methods, and potential confounding factors). Despite these limitations, this review adds to the emerging evidence that men’s health before conception may be associated with infant birth weight. Further experimental studies are necessary to assess causality.

## Conclusion

This scoping review provides a comprehensive overview of paternal preconception health and its influence on infant birth weight, accounting for physical, behavioral, and environmental factors. Factors such as paternal BMI, medical conditions, certain medications, and environmental exposure to toxic agents were found to be significantly associated with infant birth weight. These findings challenge the traditional emphasis on mothers in preconception care and suggest that more focus should be placed on improving paternal preconception health and raising awareness regarding its importance. Subsequent research on paternal preconception health should further explore the impact of mental health, body composition, and drug consumption. This could contribute to shrinking the research gaps identified in the present review, providing essential insights into how fetal and infant health can be enhanced through the provision of preconception healthcare.

## Supporting information

S1 Table Medline Search Strategy. (DOCX)

S2 Table PRISMA ScR Checklist. (DOCX)
