# Supplementary material for: The influence of paternal preconception health on infant birth weight: A scoping review
Source: PLoS One. 2026 Mar 13;21(3):e0344275. doi: 10.1371/journal.pone.0344275 (PMC12987490; doi:10.1371/journal.pone.0344275)
Supplement: S1 Table — (DOCX) [file pone.0344275.s001.docx]

### S1 Table: Search strategy for Medline

Database: MEDLINE All (Ovid)

Date of search: June 30, 2024

## MEDLINE All (Ovid)

Date: January 16, 2024, updated June 30, 2024

| **#** | **Searches** |
| --- | --- |
| 1 | (preconception or pre-conception or periconception or peri-conception or prepregnan* or pre-pregnan* or interconception or inter-conception).ti,ab,kf. |
| 2 | (before adj1 (conception or conceiv* or pregnan*)).ti,ab,kf. |
| 3 | Preconception Care/ |
| 4 | or/1-3 |
| 5 | (father? or dad? or paternal or husband? or spous* or partner?).ti,ab,kf. |
| 6 | exp Fathers/ |
| 7 | Paternal Behavior/ |
| 8 | Spouses/ |
| 9 | or/5-8 |
| 10 | 4 and 9 |
| 11 | exp animals/ not humans.sh. |
| 12 | 10 not 11 |
